# Biomarkers for monitoring intestinal health in poultry: present status and future perspectives

**DOI:** 10.1186/s13567-018-0538-6

**Published:** 2018-05-08

**Authors:** Richard Ducatelle, Evy Goossens, Fien De Meyer, Venessa Eeckhaut, Gunther Antonissen, Freddy Haesebrouck, Filip Van Immerseel

**Affiliations:** 1 Department of Pathology, Bacteriology and Avian Diseases, Faculty of Veterinary Medicine, Ghent University, Salisburylaan 133, 9820 Merelbeke, Belgium; 2 Department of Pharmacology and Toxicology, Faculty of Veterinary Medicine, Ghent University, Salisburylaan 133, 9820 Merelbeke, Belgium

## Abstract

Intestinal health is determined by host (immunity, mucosal barrier), nutritional, microbial and environmental factors. Deficiencies in intestinal health are associated with shifts in the composition of the intestinal microbiome (dysbiosis), leakage of the mucosal barrier and/or inflammation. Since the ban on growth promoting antimicrobials in animal feed, these dysbiosis-related problems have become a major issue, especially in intensive animal farming. The economical and animal welfare consequences are considerable. Consequently, there is a need for continuous monitoring of the intestinal health status, particularly in intensively reared animals, where the intestinal function is often pushed to the limit. In the current review, the recent advances in the field of intestinal health biomarkers, both in human and veterinary medicine are discussed, trying to identify present and future markers of intestinal health in poultry. The most promising new biomarkers will be stable molecules ending up in the feces and litter that can be quantified, preferably using rapid and simple pen-side tests. It is unlikely, however, that a single biomarker will be sufficient to follow up all aspects of intestinal health. Combinations of multiple biomarkers and/or metabarcoding, metagenomic, metatranscriptomic, metaproteomic and metabolomic approaches will be the way to go in the future. Candidate biomarkers currently are being investigated by many research groups, but the validation will be a major challenge, due to the complexity of intestinal health in the field.

## Introduction

Intestinal health is crucial for the general health and well-being of animals and humans alike. In farm animals, feed intake and the efficient absorption of nutrients are very much determined by the health status of the gastro-intestinal (GI) tract. Particularly in poultry, more than 50 years of intensive selection for higher daily weight gain and lower feed conversion ratio has generated breeds that are characterized by an extremely high feed intake. Excessive amounts of feed, but also certain feed ingredients [1] may put considerable stress on the digestive system. Passed a certain threshold, even in the absence of any specific pathogens, this may damage the health status of the GI tract, leading to partial loss of function (malabsorption/diarrhea). Three different, but interconnected, mechanisms appear to be involved, each of which is the focus of intensive current research: dysbiosis, leakage of the mucosal barrier, and inflammation.

Dysbiosis is an ill-defined term referring to disruption of the gut microbiota composition accompanying intestinal inflammation [2]. Delineating when a shift in microbiota composition should be considered as dysbiosis is probably the biggest current challenge for scientists investigating the intestinal microbiome. Nevertheless, even if there appears to be no universal microbial signature of dysbiosis, at least some microbial signatures have been identified as indicative of dysbiosis in laboratory animals and in man [3]. Whether these signatures also apply to poultry is hitherto unclear.

Intestinal barrier permeability is defined as the facility with which intestinal epithelium allows molecules to pass through by non-mediated passive diffusion [4]. The passive diffusion of potentially harmful small molecules from the intestinal lumen into the epithelial cells is counteracted by a family of plasma-membrane bound efflux pumps, called ATP-binding cassette transporters or multi-drug resistance (MDR) pumps [5]. These MDR pumps seem to be expressed in the intestinal tract of all animal species including poultry [6]. A defect in the MDR pumps leads to intestinal inflammation [7]. Conversely, inflammation is associated with decreased MDR1 expression in the human intestinal epithelium [8]. Intestinal barrier permeability also depends on the stability of intercellular junctions (tight junctions, adherens junctions and desmosomes) that control the paracellular transport pathway passing in between neighboring intestinal epithelial cells. Intestinal barrier permeability can be altered by a wide range of diet-derived compounds [9] and by many enteric pathogen-derived toxins, as recently reviewed in the chicken by Awad et al. [10]. Changes in the molecular structure of the junctional complexes or reduced expression of junctional structural proteins will result in decreased absorption of nutrients, increased secretory passage of ions and water causing leak flux diarrhea (gut leakage), and increased passage of macromolecules from the lumen which may induce inflammation [11]. A direct link between intestinal inflammation and loss of intercellular junction integrity has been repeatedly observed [12, 13]. In line with these observations, the presence of pro-inflammatory cytokines as such can increase epithelial permeability in vitro [12]. As dysbiosis has been associated also with destabilization of tight junctions [14], it appears that there is an intimate link between dysbiosis, intestinal barrier disruption and inflammation. Although an exhaustive review of the different host, environmental and nutritional factors that may act as primary triggers of these disturbances of the intestinal ecosystem is outside the scope of this paper, some may be useful biomarkers and thus will be mentioned in the following sections. Dysbiosis and related GI health problems are increasing both in animals and in man, to the point that this is believed to become the major human non-communicable inflammatory disease pandemic of the 21st century [15]. In poultry, dysbiosis, intestinal barrier leakage and intestinal inflammation have become major issues especially since the ban on antimicrobial growth promoters (AGP) in animal feed in the European Union in 2006 [Regulation (EC) N° 1831/2003]. Prior to that date, it was thought that intestinal health issues were largely kept under control by the widespread use of AGP. The mode of action of these subtherapeutic levels of antibiotics is not fully explained. Nevertheless, at least one of the underlying mechanisms for some AGPs appears to be through the suppression of microbial deconjugation of bile acids [16], leading to enhanced ileal absorption of lipids and availability of α-tocopherol [17]. Other possible explanations have been reviewed [18]. It has been reported that the ban on AGP in poultry feed may lead to an increase in the therapeutic use of antibiotics (usually through the drinking water), with enteric diseases and necrotic enteritis in particular as major indications [19]. Administration of antibiotics at therapeutic dosage may, however, trigger dysbiosis. All of these considerations have fueled the search for biomarkers allowing early detection of dysbiosis-related intestinal health issues.

Biomarkers are defined as measurable alterations in biological substances that associate with normal or abnormal conditions [20]. There is a need for reliable, specific, sensitive and robust biomarkers to follow up the GI health status in poultry. They would not only facilitate studies on the pathogenesis, it would also help to monitor the situation in the field, and thereby, hopefully, build prevention strategies and reduce the need for therapeutic antibiotics. The present paper is meant not only to review the limited currently available data on biomarkers for intestinal health in chickens, but also to try and identify potential candidate markers based on data obtained in other animal species and humans.

## Biomarkers requiring invasive sampling

### Biomarkers in the intestinal wall

The single layer of epithelial cells lining the intestinal lumen is continuously renewed from the pool of crypt-based stem cells. The newly formed cells migrate up the villus, to finally enter anoikis (a special form of programmed cell death) [21] and exfoliate from the villus tip [22]. During migration, the cells differentiate and thus the cells near the villus tips are most important for nutrient absorption. Some enteric pathogens such as coccidia will directly cause epithelial cell death. Increased loss of villous epithelial cells, resulting in decreased villus length, appears to be very common in (severe) intestinal health problems [22]. It is partly compensated by increased proliferation, resulting in increased crypt depth. Therefore, simple measurements of villus height, crypt depth and the villus/crypt ratio have become the gold standard in the evaluation of the intestinal health status in animals. Villus height, crypt depth and/or villus crypt ratios, measured at the level of the duodenum, the jejunum or the ileum are widely used as the standard read-out for the evaluation of intestinal health in poultry in studies investigating the effects of feed ingredients and feed additives [23]. Reference values for broilers at 23 days in duodenum, jejunum and ileum are approximately 1400, 900 and 700 mm for villus height, 190, 170 and 160 mm for crypt depth, and 8, 6 and 5 for villus height to crypt depth ratio [24].

There is controversy in the literature about the role of epithelial oxygenation in GI tract inflammation, with some studies showing inflammation to lead to mucosal hypoxia after *Salmonella* infection in mice [25], while other studies show increased epithelial oxygenation, leading to aerobic luminal expansion of *Salmonella* in mice [26]. In vitro studies using intestinal epithelial cell lines provide evidence that mitochondrial respiration plays an essential role in the maintenance of tight junction stability as measured by TEER [27]. Inflammation-associated oxidative stress can change the phenotype of the intestinal epithelial cells resulting in changes in the expression of genes that could be used as biomarkers, as was recently shown in broilers [28]. Upregulation of expression was noted for interleukin 8, interleukin 1, transforming growth factor-β4 and fatty acid-binding protein 6, whereas fatty acid-binding protein 2, occludin and mucin 2 were downregulated.

Diet-related, either or not microbiota-derived, metabolites engage the upregulation of metabolite-sensing G-protein-coupled and other receptors on intestinal epithelial cells, as shown in laboratory animals [29]. Such biomarkers have not been used in chickens so far. Nevertheless, similar receptors undoubtedly are expressed also on the chicken intestinal epithelium, and thus, quantification of these receptors in intestinal biopsies could be useful for evaluation of intestinal health. In lab animals it has been shown also that dysbiosis and/or the presence of facultative pathogenic microorganisms may trigger a shift in phenotype of the epithelial cells with increased expression of defense molecules such as intestinal alkaline phosphatase [30], which could represent a candidate negative marker of intestinal health also in poultry.

Next to the absorptive epithelial cells, a number of other cell types in the intestinal mucosa could also carry useful biomarkers. It has been shown that enteroendocrine cell density can be influenced by the diet in case of intestinal inflammation in humans [31]. Similarly, in the chicken, increased enteroendocrine L-cell density in the ileal mucosa was observed in parallel to other positively responding intestinal health parameters in a study using an enzymatically treated wheat extract as a prebiotic [32].

The healthy propria mucosae will contain a high number of tolerogenic FoxP3-positive regulatory T-lymphocytes (Treg), whereas these Treg have been shown to be deficient in inflammatory bowel disease in humans [33]. Measuring Treg density in intestinal mucosal biopsies may represent a valuable criterion for intestinal health, which could be of use also in the chicken. Conversely, neutrophil granulocyte influx in the lamina propria has been shown to be increased in mouse models, but not in chicken models of intestinal permeability defects [34]. In the chicken, total T-lymphocyte counts in the lamina propria mucosae has been evaluated in several studies. It is usually found to parallel villus shortening [23, 32].

### Biomarkers in blood and in liver

Increased numbers of bacteria passing through damaged tight junctions of the intestinal epithelium (bacterial translocation) can reach the liver and induce inflammation [35]. In response (the so-called acute phase response), the secretion of proteins by the hepatocytes changes. These acute phase proteins can be measured in serum. In man, the clinical form of chronic, non-pathogen-induced intestinal inflammation, classified under the common denominator of inflammatory bowel disease (IBD), has been associated consistently with an acute phase response in the liver, resulting in a significant increase in acute phase proteins (especially C-reactive protein and lipopolysaccharide-binding protein (LBP)) in serum [36, 37]. In the chicken three different experimental models have been used to increase the permeability of the intestinal mucosal barrier, but the effect on the intestinal barrier was insufficient to observe any changes in serum acute phase proteins [38, 39]. Moreover, an acute phase response in the liver can also be seen in response to any inflammatory process, also to one taking place in other parts of the body, outside the intestinal tract, so one can doubt about the specificity of serum acute phase proteins as markers of (poor) intestinal health [40].

Increased intestinal permeability is associated with more bacteria from the gut reaching the bloodstream and the liver. Therefore bacterial counts in the liver have been used as biomarker for increased intestinal permeability in broilers [41] and in turkeys [42]. Leaking epithelial junctional complexes will also allow passage of bacteria-derived macromolecules, such as lipopolysaccharide (LPS) from gram-negative bacteria, as has been shown in a nutritional/coccidiosis model of intestinal barrier leakage in broilers [28]. In the healthy intestine, LPS is not leaking through the paracellular pathway. It is internalized in the epithelial cells and detoxified by the epithelial cell alkaline phosphatase [43]. Consequently, the detection and quantification of LPS in serum could be an elegant indicator of increased paracellular permeability. Unfortunately, most techniques to measure LPS are not very reliable.

d-lactate is one of the numerous metabolites produced by intestinal bacteria. Although it can be further metabolized by the bacteria, in animals and humans with increased intestinal barrier permeability, elevated d-lactate concentrations can be measured in serum, probably as a consequence also of excess intestinal microbial production [44]. It has been used in chickens [45] as a serum biomarker of intestinal permeability but also depends on the concentrations available in the gut and these can vary.

Finally, the damage to the epithelial cells can result in the release of certain intestinal epithelial cell-specific proteins into the blood stream. This has been reported for the enzymes diaminoxidase in chickens [45] and intestinal fatty acid binding protein 6 in an experimental broiler model of intestinal barrier disruption [28], although the latter was not confirmed in other experimental broiler studies [38, 39]. In the latter studies, only tight junction protein concentrations were increased in plasma in a 19.5 h fasting experiment.

## Biomarkers allowing non-invasive sampling

### Fecal microbiota as biomarkers

The advent of next generation sequencing technology has allowed studying the composition of the intestinal microbial community in different animal species including poultry. The most widely used phylogenetic marker is the bacterial 16S small subunit ribosomal RNA gene, which has both conserved and variable regions and which is universally present in prokaryotes. These high throughput technologies allow the collection of large sets of data. These data, however, should be interpreted with care. Indeed, even if considerable efforts are being made to standardize analytical procedures [46], standardization is still an issue and technical artefacts do occur. Also, the depth of analysis of amplicon sequencing could be improved and species taxonomic assignation of OTUs (operational taxonomic units) is far from being precise. Nevertheless, microbiota patterns reliably associated with poor intestinal health are currently being identified in man and in animals, including chickens.

In the chicken the microbiota is quantitatively and functionally most developed in the ceca. Fecal microbiotas are often used as proxy for the intestinal microbiota. Even if it has been shown in the chicken that the fecal and cecal microbiota are qualitatively similar, there are quantitative differences within the different bacterial groups [47]. Therefore, fecal microbiota analyses should be interpreted with care, not only in the chicken, but also in other animals and in humans. Nevertheless, in man much effort has been done to identify fecal microbiota patterns associated with IBD [48]. One characteristic pattern of the intestinal microbiota that appears to be constantly associated with many forms of poor intestinal health both in animals and in man is loss of species richness, and/or diversity and evenness [49]. Unfortunately, these parameters are very difficult to measure. Indeed, there is even considerable confusion about the normal species richness in the healthy human gut microbiota, with numbers varying from 100 to 1000 species [50]. Therefore, many research groups have been hunting for more specific microbial signatures of dysbiosis. One such signature appears to be the loss of bacterial groups belonging to the phylum *Firmicutes*, as has been shown in human Crohn’s disease [51]. The *Firmicutes,* however, constitute a heterogeneous phylum containing bacterial groups with different metabolic activities, which makes a change at the phylum level a less powerful indicator. Nevertheless, several studies on beneficial prebiotics in broilers have shown expansion of *Firmicutes* [32] or higher *Firmicutes* to *Bacteroidetes* ratios [52].

Within the butyrate-producing *Firmicutes*, more specific markers have been found in specific pathological entities, such as decreases in *Roseburia hominis* and *Fecalibacterium prauznitzii* [53] in people with ulcerative colitis. The mucosa-associated *Butyricicoccus* genus is not only decreased in people with ulcerative colitis [54], it is also proposed as a biomarker for healthy mucosa-associated microbiota in man [55]. This same *Butyricicoccus* was shown to support intestinal health after administration as a probiotic in broilers [56]. There appears to be a consensus in the literature that intestinal inflammation supports the expansion of facultative anaerobic bacteria [57]. More specifically, the expansion of the phylum *Proteobacteria* has been proposed as a diagnostic signature of dysbiosis in man [3]. Within this phylum, the outgrowth of the *Enterobacteriaceae* family in particular has been denoted as signature of inflammation-associated dysbiosis in the mouse model [2]. In the chicken, a negative correlation between performance parameters (as read out for intestinal health) and *Enterobacteriaceae* expansion has been reported [56]. Therefore, quantification of *Enterobacteriaceae* using Q-PCR or other means may be of use to measure dysbiosis in poultry. Next to the *Enterobacteriaceae*, also the expansion of the sulfate reducing *Desulfovibrio* genus has been noted as a signature of IBD in humans [48]. Both *Enterobacteriaceae* and *Desulfovibrio* are potential sulfate reducers. Their expansion can lead to the excessive production of toxic concentrations of hydrogen sulfide.

In the healthy gut, flagellin-specific IgA together with innate immune mechanisms appear to quench expression of flagellin protein in lab animal experimental studies [58]. As a consequence, levels of flagellin protein are low in the healthy gut in humans [59], whereas mucosal barrier breakdown and inflammation in the human gut have been associated with high levels of flagellin in the intestinal lumen [60]. Although it was not reported to be used so far in the chicken, flagellin might represent a valuable candidate biomarker of dysbiosis in the chicken.

### Fecal biomarkers of microbiota metabolism

The microbiota converts complex (non-starch) carbohydrates and fibers, as well as proteins, into a range of terminal metabolites that can have diverse effects on host health [61]. Diet not only impacts on the microbiota composition but also on microbiota metabolism, which in turn can impact on host health (reviewed in the human host in [62]).

Terminal metabolites produced from non-starch polysaccharides are predominantly the short chain fatty acids propionate and butyrate. The multiple beneficial effects of butyrate have been extensively investigated (for review see [63]). Also propionate is known to have beneficial effects on host health. Thus concentrations of butyrate and propionate in feces or in intestinal content might be valuable indirect indicators of intestinal health. However, as butyrate and propionate are taken up by the intestinal epithelium through receptor mediated processes, the concentration in the lumen depends on the balance between microbial synthesis and mucosal absorption. One way around this may be by quantifying the microbial capacity of butyrate production using Q-PCR to evaluate the number of gene copies encoding a key bacterial enzyme in the main butyrate production pathway, the butyryl-CoA:acetate CoA transferase [64]. In one experimental study in broilers, this biomarker was shown to be associated with improved intestinal health [65].

In man it is well established that high protein, low carbohydrate diets alter the colonic microbiota, favoring a potentially pathogenic and pro-inflammatory microbiota [66]. Terminal metabolites produced from proteins and peptides include a range of amines, thiols, indoles, phenols and branched chain fatty acids, but also a number of volatile compounds such as hydrogen sulfide and ammonia [61]. Most of these metabolites appear to have beneficial effects on the intestinal barrier when produced in low quantities, but harmful effects when produced in larger quantities, as was recently reviewed also in the chicken [67]. For instance, a low concentration of hydrogen sulfide was shown to protect from colitis in an experimental mouse model [68]. Conversely, reduction of hydrogen sulfide production in the colon by restricting the intake of food rich in sulfur amino acids appears to be beneficial in human patients with ulcerative colitis [69].

Of particular importance for intestinal health is the above mentioned inflammation-associated expansion of the *Enterobacteriaceae* family. In the mouse model, this was shown to be accompanied by elevated formate concentrations in the gut [2]. Further in the mouse model, the oxygen radicals generated during inflammation were shown to react with sulfur compounds present in the intestinal lumen to form tetrathionate, which in turn favors the expansion of *Salmonella* [70] and *Campylobacter* [71]. To the best of our knowledge, none of these have been evaluated in poultry so far.

In man, changes in volume or composition of fecal protein-derived (hydrogen sulfide, ammonia) or other (hydrogen, carbon dioxide, methane) volatile compounds have been proposed as useful biomarkers of intestinal health [72, 73]. Changes in volumes of these compounds indicate a shift in the microbiota composition. Investigations of poultry volatile fecal compounds so far were focusing on their contribution to malodourous environmental pollution. Nevertheless, these biogases also hold promise as novel markers of intestinal health in the animals. Simple devices are available for analyzing biogases in air samples and for some biogases colorimetric assays are available. This is an area that merits further investigation.

## Host factors as fecal biomarkers

All higher organisms have evolved complex interactions with their gut microbiome and have developed mechanisms to maintain homeostasis in the gut, mostly by secreting factors that regulate the luminal microbiota but also by adapting the composition of the outer mucus layer where a select population of microorganisms is allowed [74]. When the conditions in the gut lumen change, or in case of dysbiosis or intestinal inflammation, a range of host proteins will be secreted, released or lost into the intestinal lumen. Especially when these proteins are stable and resist enzymatic degradation, they can be very useful biomarkers of intestinal inflammation. A number of these proteins have been used as biomarkers for early detection of flare-ups and for the follow-up of therapy response in human patients with IBD. Calprotectin, a protein derived from the granules of neutrophilic granulocytes, is routinely measured in stool samples of these patients. Unfortunately, even if calprotectin is a highly conserved protein, it is not expressed in the chicken heterophilic granulocyte. Next to calprotectin, other defense molecules, such as lactoferrin, cathelicidins and defensins may be secreted in increased amounts in case of damage to the intestinal epithelial barrier [75]. Many other host factors have been proposed for use in human patients, as reviewed by Lewis [76]. None of these are commonly in use for poultry.

Under conditions of intestinal epithelial damage, peptidases and other enzymes secreted predominantly by highly differentiated epithelial cells will be reduced in concentration. So these could also be valuable biomarkers of intestinal health. Moreover, when intestinal epithelial cells are injured and lysed, intracellular compounds can be released and end up in the feces. One such parameter could be Zn^2+^, as released from the intracellular zinc stock [77]. This whole area is under investigated in poultry.

## Conclusions

There is a high demand for precocious, simple and reliable biomarkers of intestinal health in poultry. A number of quantifiable biomarkers of intestinal health are already available for use in poultry today (Table 1).

**Table 1:** **Currently available quantifiable biomarkers for evaluation of intestinal health in poultry**

Source	Reference values available
Intestinal tissue biopsies	
Villus length	Yes [30]
Crypt depth	Yes [30]
Villus/crypt ratio	Yes [30]
L-cell density	No
T-lymphocytes in propria mucosae	No
Blood and liver	
Total bacterial count in liver	No
d-lactate in blood	No
Diaminoxidase in blood	No
Caecal content/faeces	
Firmicutes	No
Enterobacteriaceae	No
Acetate-CoA butyrate-CoA transferase/bisulfite reductase Q-PCR	No
Butyrate	No

**Figure 1:**
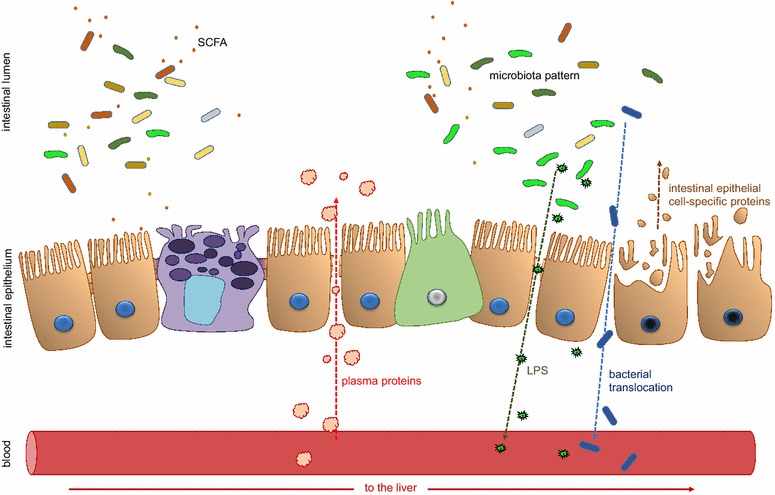
**Intestinal mucosal barrier damage.** Different aspects of intestinal health defects (leakage inside-out/outside-in; dysbiosis; inflammation) can be evaluated in different types of samples (blood; liver; mucosal biopsies; intestinal content; faeces).

Most currently available biomarkers, however, have a number of disadvantages, so there is room for improvement. In this paper, we have classified the different markers depending on their source and the sample type in which they can be detected (Figure 1).

In all cases of disturbance of intestinal health, the intestinal mucosa is where the damage is taking place, so biopsies of the intestinal mucosa provide the most reliable markers. Unfortunately, the processing of these samples and the analytical techniques are rather sophisticated and slow, therefore there is a demand for alternative biomarkers. These proxies need to be benchmarked against the well-established biopsy-based criteria. The most commonly used gold standard of intestinal mucosal damage is the villus length, crypt depth and villus/crypt ratio. This morphological parameter will, however, only change in case of pathological epithelial cell death. Dysbiosis and leakage of the paracellular pathway may be present in the absence of increased epithelial cell loss. Novel biomarkers thus may be more sensitive and more precocious than the traditional histological markers. The most promising new biomarkers will be stable molecules ending up in the feces and litter that can be easily quantified, preferably using rapid and simple pen-side tests. It is unlikely, however, that a single biomarker will be sufficient to follow up all aspects of intestinal health and deficiencies thereof. Combinations of multiple biomarkers will be the way to go in the future. Holistic approaches, such as amplicon sequencing, providing information on all shifts in the microbiota, will soon become sufficiently cost-effective and fast to be used for the follow-up of the intestinal health status of poultry in practice. On a longer term, also metagenomics, metatranscriptomic, metaproteomic and metabolomic approaches in combination with machine learning, will be powerful tools for the design of algorithms that will allow continuous monitoring of the intestinal health status.

## Abbreviations

GI: gastrointestinal
TEER: trans-epithelial electrical resistance
IBD: inflammatory bowel disease
LBP: lipopolysaccharide-binding protein
LPS: lipopolysaccharide
MDR: multidrug resistance
OTU: operational taxonomic unit
Q-PCR: quantitative polymerase chain reaction
